# Fragmented Chemotherapy of Plasmablastic Lymphoma in an Immunocompromised Patient

**DOI:** 10.7759/cureus.88102

**Published:** 2025-07-16

**Authors:** Sabrina E Chin, Ellen A Wood, Mandelise N Laudat, Rodrigo Santoscoy-Valencia, Ilya Fonarov, Damian Casadesus

**Affiliations:** 1 Internal Medicine, Jackson Memorial Hospital, Miami, USA; 2 Pathology and Laboratory Medicine, Jackson Memorial Hospital, Miami, USA

**Keywords:** aids, cd138, ebv (epstein-barr virus), hiv lymphoma, human immunodeficiency virus (hiv), immunocompromised patient, myc gene rearrangement, plasmablastic lymphoma (pbl), plasma cell, r-epoch (rituximab

## Abstract

Plasmablastic lymphoma (PbL) is an uncommon subtype of diffuse large B-cell lymphoma, which is closely associated with human immunodeficiency virus (HIV) and Epstein-Barr virus* *(EBV) infections. Due to its rarity and characteristically aggressive nature, it has proven difficult to treat.

A rapidly growing mandibular mass can raise suspicion for malignancy. Our patient presented with a painful, enlarging mandibular mass in the absence of fever, night sweats, or fatigue. A review of imaging showed an 8.1 x 5.6 x 9.4 cm central destructive mass with soft tissue swelling, facial skin thickening, and involvement of facial bones. Biopsy was indicative of PbL. The patient was administered a chemotherapy regimen. However, the patient eloped during initial inpatient treatment. The patient returned to the hospital, but subsequent chemotherapy dispensing was irregular and incomplete. Due to the late presentation of the patient and fragmented treatment, the long-term prognosis is poor.

## Introduction

Plasmablastic lymphoma (PbL) can present as an enlarging mass. PbL is characteristically aggressive in nature, and the presence of viral protein expression, as well as its preference for the oral cavity, makes it resemble other lymphomas such as Burkitt’s lymphoma, complicating diagnosis [1]. PbL is presumed to originate from the plasmablast, a B-cell subtype that has experienced class-switching and somatic hypermutation [2]. The immunophenotype is typically characterized by positive expression of B-cell markers CD79a, IRF-4/MUM-1, BLIMP-1, CD38, and CD138, and negative for B-cell markers such as CD20 and PAX-5 [2]. It typically presents with extranodal involvement, and an analysis of 481 PbL cases in the National Cancer Data Base (NCDB) found that the most common sites are the gastrointestinal tract (34%), head and neck (27%), skin or connective tissue (16%), and bone marrow (7%) [3]. While cases have been documented in immunocompetent patients, PbL demonstrates a close association with HIV and EBV infections [4]. Most cases have been seen in the age range of 42 to 55, with an incidence showing a predominance in male HIV-positive patients and female HIV-negative patients. Treatment of PbL has proven difficult given its rarity and aggressive, relapsing clinical course. However, six to eight cycles of dose-adjusted etoposide, vincristine, doxorubicin, cyclophosphamide, and prednisone (EPOCH) are suggested by current guidelines, with intrathecal chemotherapy being administered in select cases [5]. In patients with HIV, anti-retroviral therapy should begin or be continued throughout PbL treatment. Surgical intervention has no role, and radiation oncology is rarely used in the initial treatment for PbL [5]. Without early detection and management, the prognosis is poor, with a median overall survival (OS) for HIV-positive patients being three months and for HIV-negative patients being four months [4].

## Case presentation

**Figure 1:**
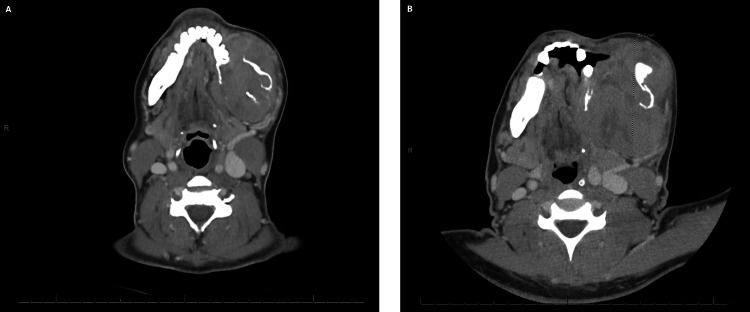
Axial computed tomography (CT) with contrast of an expansile mass centered in the left mandibular body A) The mass during a previous hospital visit one month prior measuring 5.7 x 6.1 x 8.2 cm (AP by TV by CC); B) The mass at time of presentation measuring 8.1 x 5.6 x 9.4 cm (AP by TV by CC) AP: anteroposterior; TV: transverse; CC: craniocaudal

A female patient in her 40s with a history of HIV and bipolar disorder presented to the emergency department (ED) with a painful and enlarging left mandibular mass. The mass had been present for three months and was associated with ipsilateral loose teeth. When compared to a computed tomography (CT) of the face taken one month prior, progressive growth from 5.7 x 6.1 x 8.2 cm to 8.1 x 5.6 x 9.4 cm was recorded (Figures 1A, 1B). The patient had no fevers, weight loss, night sweats, or fatigue. She smoked cigarettes and had a history of remote methamphetamine usage. Her medication included a combination of bictegravir, emtricitabine, tenofovir, and, additionally, aripiprazole and citalopram. She had a known drug allergy to Bactrim (trimethoprim-sulfamethoxazole).

**Figure 2:**
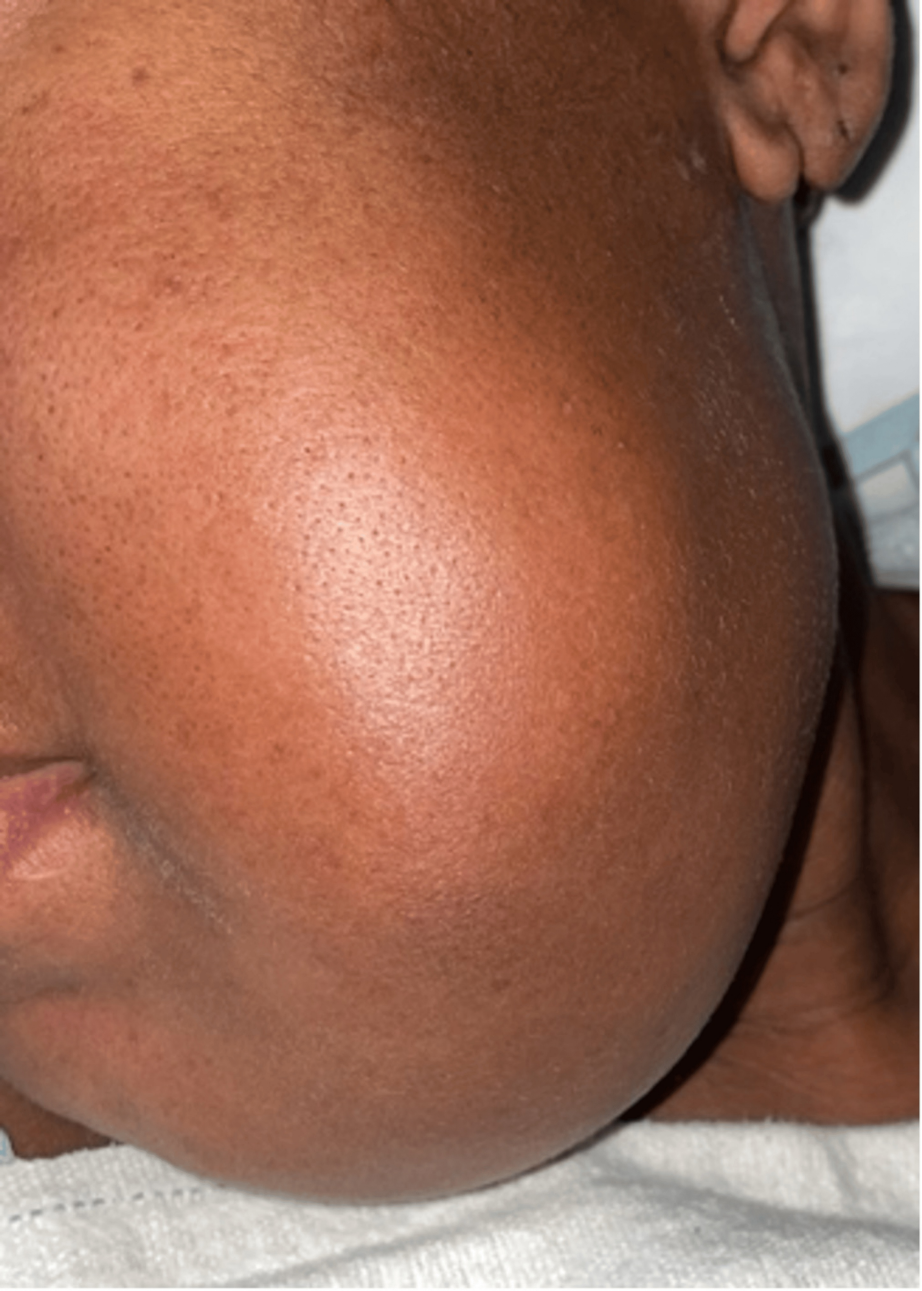
Left mandible enlargement with no skin discoloration or variability from the rest of the facial skin

**Figure 3:**
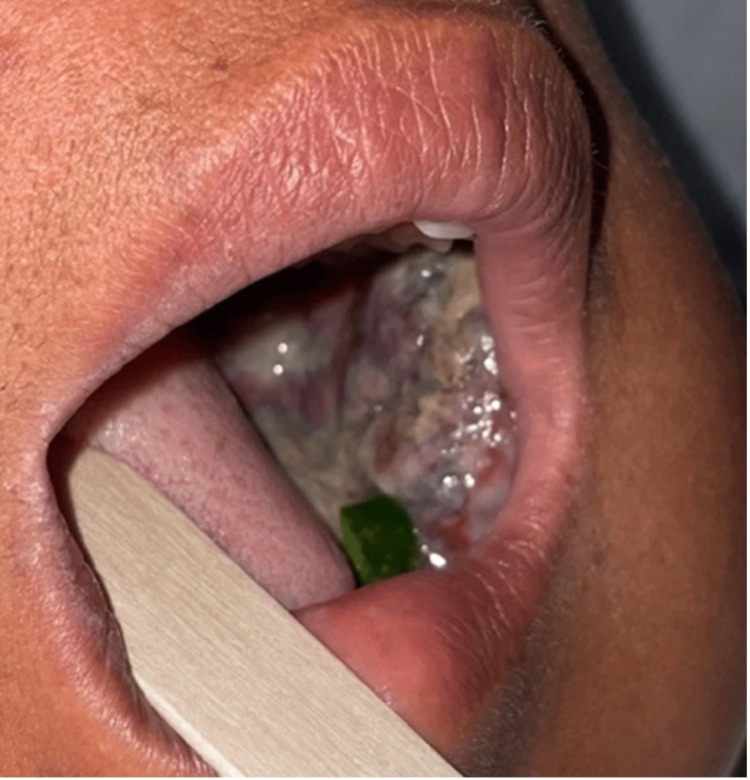
A 'fleshy' mass occupying the entirety of the left cheek and gum with friable mucosa

On physical exam, the vital signs were blood pressure of 108/74 mmHg, temperature of 37.2 °C, heart rate of 78 beats per minute, and respiratory rate of 19 breaths per minute. A visible unilateral enlargement of the left mandible was observed with no skin discoloration or variability from the rest of the facial skin (Figure 2). The oral cavity revealed poor dentition, including multiple dental caries and broken teeth. There was a fleshy mass occupying the entirety of the left cheek and gum with friable mucosa and episodic bleeding (Figure 3). The range of motion of the left mandible was limited, and the sensation on the lower left face was diminished. The remainder of the head and neck, cardiovascular, pulmonary, and abdominal exam was unremarkable.

Investigations

**Figure 4:**
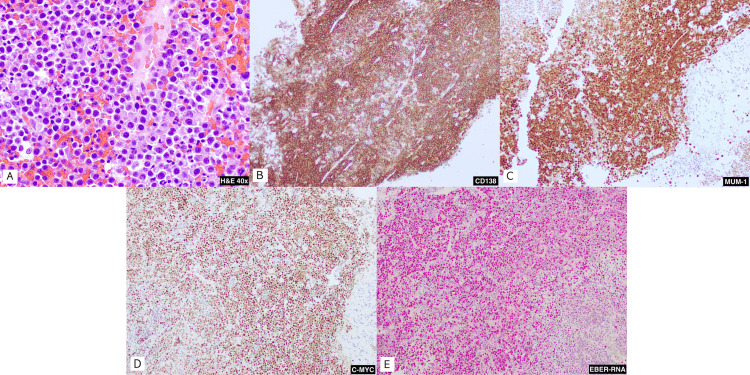
Histopathology A. (40x) Soft tissue composed of sheets of lymphoma cells with plasmablastic morphology, including eccentrically placed nuclei, with open chromatin and prominent nucleoli; B. Immunohistochemical staining shows CD138 positivity; C. Immunohistochemical staining shows MUM-1 positivity; D. Immunohistochemical staining shows MYC positivity; E. EBV encoded RNA in situ hybridization positivity

Lab testing revealed a glucose of 138 mg/dL (74 - 106 mg/dL), blood urea nitrogen (BUN) of 20 mg/dL (7 - 17 mg/dL), and bicarbonate of 33 mmol/L (22 - 30 mmol/L). EBV quantitative was 19000 IU/mL, and an absolute CD4 count of 156.88 cells/mcL (490 -1740 cells/mcL) with an undetected HIV-1 RNA quantitative PCR. A CT scan of the head and neck demonstrated an 8.1 x 5.6 x 9.4 cm central destructive mass with soft tissue swelling and facial skin thickening, as well as involvement of the mass within facial bones (Figure 1B). The biopsy of the left mandible mass showed lymphoma cells with plasmablastic morphology (Figure 4A). Immunohistochemistry was positive for CD138, MUM-1, CD45 (weak), and MYC (80%) (Figures 4B-4D). EBV-encoded RNA in situ hybridization was positive (Figure 4E). Immunohistochemistry was negative for CD20, CD79a, Pax-5, ALK and HHV8. Lumbar puncture was performed, and the cerebrospinal fluid cytology and flow cytometry were negative for malignant cells or markers. A positron emission tomography (PET)/CT was not performed. A CT scan of the chest was negative for metastasis. A CT scan of the abdomen and pelvis with contrast revealed a left adnexal mass, as well as concentric wall thickening of the stomach, duodenum, and proximal jejunum. The patient underwent upper endoscopy, and the biopsy of the gastric thickening was negative for malignancy. Ann Arbor staging was suspected to be stage IV due to the presence of a neck mass and an adnexal mass, but biopsy of the adnexal mass was not recommended due to concerns of seeding. The patient was recommended to have surgical resection of the adnexal mass.

Outcome and follow-up

The patient was treated with intrathecal (IT) chemotherapy with methotrexate, cytarabine, and hydrocortisone. Allopurinol and IV fluids were given to prevent tumor lysis syndrome (TLS), and acyclovir was used as prophylaxis against HSV infection. However, the patient eloped during the inpatient treatment; therefore, biopsy of the adnexal mass was not performed, and outpatient chemotherapy with daratumumab + the EPOCH regimen (etoposide, prednisone, vincristine, cyclophosphamide, doxorubicin) was unable to be scheduled.

The patient returned to the ED 15 days after elopement, complaining of facial pain, and was admitted. Lab testing revealed hemoglobin (Hb) of 6.5 g/dL (11.1-14.6 g/dL), hematocrit (Hct) of 21.6% (33.2-43.4%). A CT scan of the head and neck demonstrated a decrease in mass size to 5.2 x 7.2 x 8 cm. Due to previous elopement, the patient was not an ideal candidate for the trial of daratumumab-EPOCH treatment. It was decided that the patient would receive six cycles of rituximab-EPOCH (R-EPOCH) and IT chemotherapy with each cycle. During this visit, the patient received one dose of IT chemotherapy (methotrexate, cytarabine, hydrocortisone) and then completed cycle one (C1) of R-EPOCH over a five-day period. Hospital course complicated by methicillin-sensitive Staphylococcus aureus (MSSA) bacteremia. A definitive tissue diagnosis of the left adnexal mass was deemed not urgent at this visit. The patient was discharged with an IV PICC line for treatment with cefazolin. Follow-up appointments with Oncology and a primary care physician (PCP) were made, but the patient was a no-show.

C2 was delayed because the patient did not attend the hematology clinic. C2 was provided during an admission for intractable cancer-related pain. Initial lab testing revealed Hb of 9.5 g/dL (11.1-14.6 g/dL), Hct of 31.1% (33.2-43.4%). C2 of R-EPOCH was administered over five days, consisting of R-EPOCH given via IV over five days. After completing C2, the patient was given a dose of IT chemotherapy. Appointments were made for follow-up with Palliative Care, but the patient was a no-show.

C3 was delayed because the patient did not attend the hematology clinic. C3 was provided during an admission for intractable cancer-related pain. Initial lab testing revealed a white blood cell (WBC) count of 3.5 x 10^3^/mcL (4.0-10.5 x 10^3^/mcL), a red blood cell (RBC) count of 5.7 x 10^6^/mcL (3.80-4.90 x 10^6^/mcL), and a platelet count of 430 x 10^3^/mcL (140-400 x 10^3^/mcL). A CT scan of the head and neck demonstrated a decrease in mass size to 4.4 x 6.2 x 4.6 cm. C3 of R-EPOCH was administered over five days, and the patient received a dose of IT chemotherapy. One dose of filgrastim due to an absolute neutrophil count (ANC) of 1.9 x 10^3^/mcL (2.0-6.0 x 10^3^/mcL) was administered at the time of discharge. Follow-up appointments with Hematology, Palliative Care, and for the administration of C4 were made before discharge.

C4 was administered on time during an admission for intractable cancer-related pain. Initial lab testing revealed WBC count of 1.9 x 10^3^/mcL (4.0 - 10.5 x 10^3^/mcL), RBC count of 4.95 x 10^6^/mcL (3.80 - 4.90 x 10^6^/mcL), ANC of 1.1 x 10^3^/mcL (2.0 - 6.0 x 10^3^/mcL). A CT scan of the head and neck demonstrated a decrease in mass size to 3.8 x 4.8 x 5.8 cm. C4 of R-EPOCH was administered over five days. IT chemotherapy was attempted but was aborted due to significant resistance during the attempt at lumbar puncture. The patient was discharged with PEG filgrastim, dexamethasone, and oxycodone. Prior to discharge, follow-up with the outpatient Hematology-Oncology clinic was scheduled to administer the dose of IT chemotherapy and for the administration of C5 and C6.

The patient did not attend the oncology clinic for the dose of IT chemotherapy. Scheduled administrations of C5 and C6 were missed because of patient incarceration. Fifty-five days after the administration of C4, the patient was readmitted for possible coordination for chemotherapy after presenting to the ED for left jaw pain and dizziness. A CT scan of the head and neck demonstrated a decrease in mass size to 4.1 x 4.2 x 5.4 cm. The administration of C5 was not done during this visit. A follow-up appointment with Hematology was made prior to discharge, but the patient did not attend.

**Figure 5:**
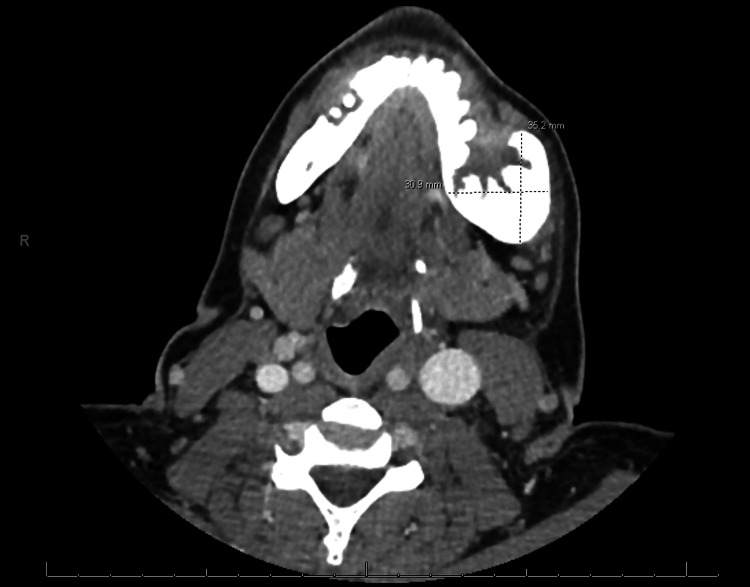
Axial CT with contrast showing an solid expansile mass centered in the left mandibular body now measuring approximately 3.5 x 3.1 cm (AP by TV) with similar local mandibular destruction and heavily calcified components primarily within the posterior and inferior aspect of the lesion AP: anteroposterior; TV: transverse

**Figure 6:**
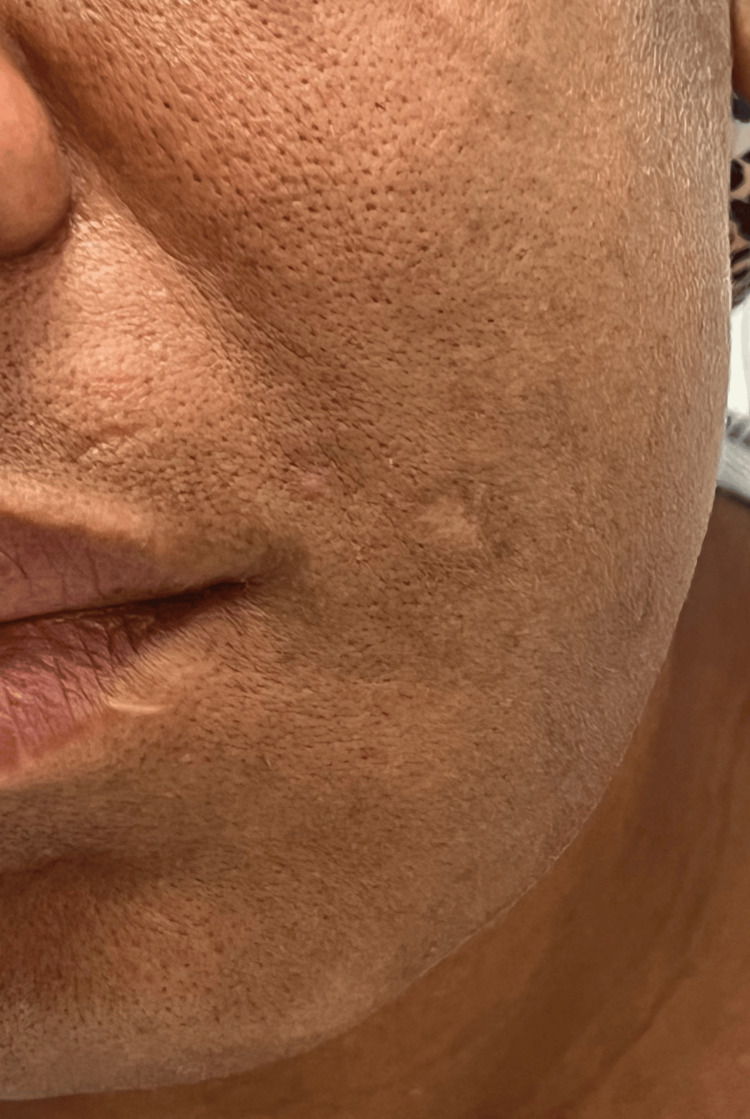
Reduced size of the left-sided facial mass with no skin discoloration or variability from the rest of the facial skin

**Figure 7:**
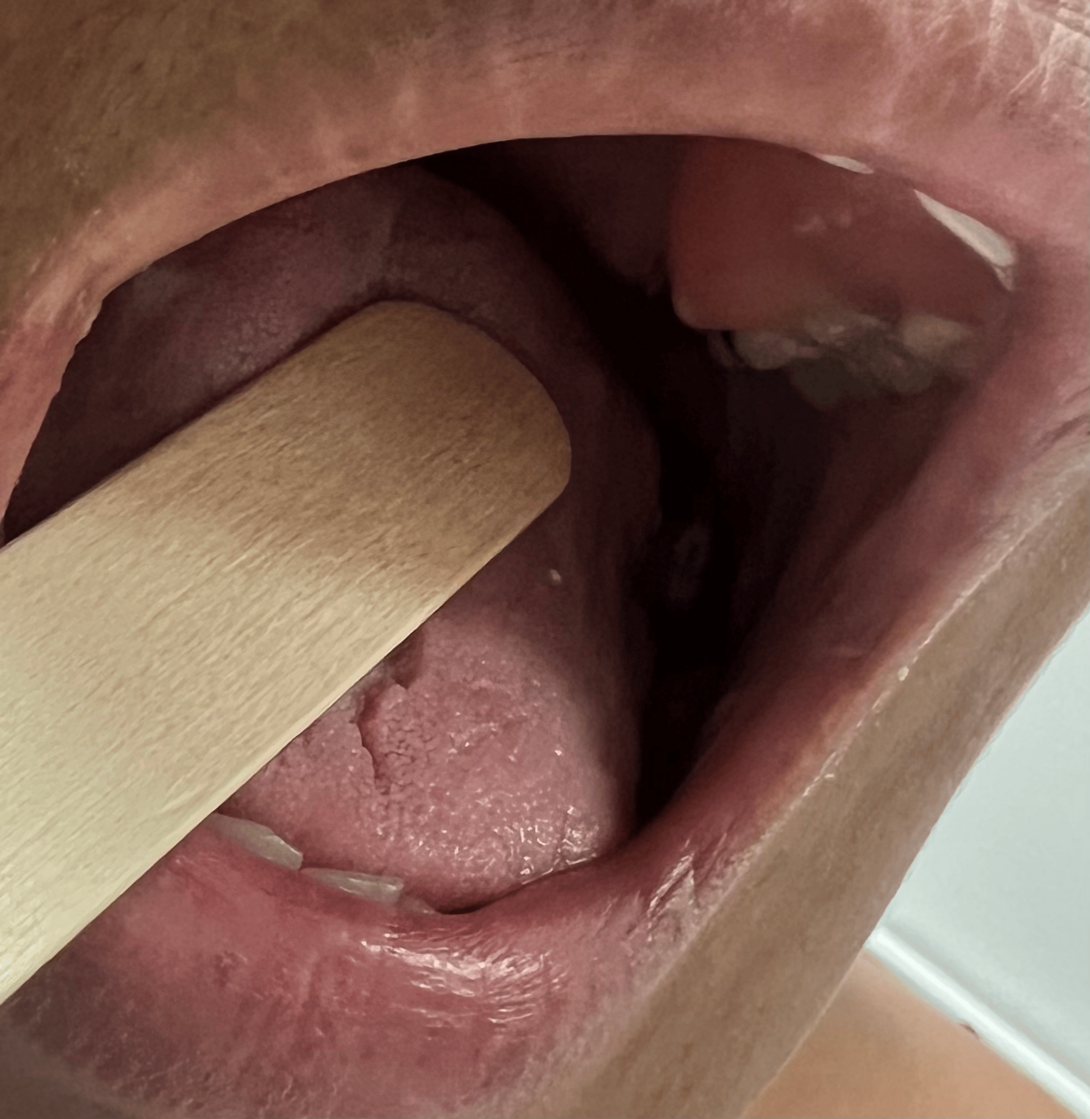
Previously observed 'fleshy' mass not readily visible on inspection of the oral cavity 15 months later

Eight months later, the patient was admitted for the administration of IV acyclovir after presenting to the ED for an acute episode of herpes zoster. During these eight months, she was disengaged from oncological care but did make two visits to the ED for jaw pain. A CT scan of the head and neck during this admission demonstrated a decrease in mass size to 3.5 x 3.1 cm (Figure 5). Inspection of the jaw showed a left-sided facial mass extending inferiorly toward the left neck with no skin discoloration or variability from the rest of the facial skin (Figure 6). The oral cavity revealed poor dentition with multiple missing teeth. The mass was not readily visible on inspection of the oral cavity (Figure 7). The administration of C5 was not done during this visit. The patient was treated with IV acyclovir for the episode of herpes zoster and discharged with follow-up appointments with Palliative Care.

## Discussion

Among other facial masses and lesions, to establish the correct diagnosis of PbL, the proper recognition of its clinical features, localization, and genetic markers is critical for patient prognosis and treatment [6]. PbL usually presents as an enlarging mass in the neck. There are multiple benign and malignant conditions with similar presentations, and patients can delay medical attention for this reason. PbL must be distinguished from other CD20-negative B-cell neoplasms, such as primary effusion lymphoma and anaplastic lymphoma kinase-positive large B-cell lymphoma, as they also display plasmablastic morphology. Commonly, PbL is associated with EBV and MYC gene rearrangements. It is also characterized by CD38, CD138, MUM1/IRF4, BLIMP1, and XBP1 plasmocytic differentiation markers [7]. Similarly to our patient, PbL has been readily described in HIV-positive, immunocompromised, and solid organ transplant patients. More recently, its prevalence in immunocompetent patients has been well-documented, though there is more variability in the anatomical distribution than in immunocompromised patients [6,8]. Even so, HIV and AIDS patients have an increased risk of developing lymphoproliferative disorders such as PbL. Commonly reported sites of PbL are within the oral cavity, but growth has been seen in nasal, paranasal, GI, skin, and soft tissue, as well as lymph nodes [8].

Treatment options include CHOP (cyclophosphamide, doxorubicin, vincristine, prednisone), hyper-CVAD-MA (hyper-fractionated cyclophosphamide, vincristine, doxorubicin, dexamethasone, and high-dose methotrexate and cytarabine), CODOX-M/IVAC (cyclophosphamide, vincristine, doxorubicin, high-dose methotrexate/ifosfamide, etoposide, and high-dose cytarabine), and EPOCH. EPOCH has shown success for aggressive lymphomas and is recommended for PbL treatment, which was used in the presented patient [2].

PbL patients have proven to be a challenge to treat. Despite a good initial response to available therapy, the rate of recurrence is high, and the prognosis remains poor. Studies have shown that patients with a history of AIDS with PbL can be treated with bortezomib and dose-adjusted EPOCH (V-EPOCH) and intrathecal chemotherapy [9]. In a specific study, a patient with PbL-positive pulmonary nodules, retroperitoneal and pelvic adenopathy, as well as a nasopharyngeal mass linked to PbL, was treated with six cycles of V-EPOCH. Post-treatment, a complete response and no PET-avid fluorodeoxyglucose uptake was observed [9]. Subsequently, research on different treatment methodologies is being done. For example, daratumumab, an anti-CD38 human IgG1 monoclonal antibody, in combination with EPOCH, can be used for six cycles. Other treatment options include lenalidomide or pomalidomide, which are immunomodulators, as well as Brentuximab vedotin, an antibody-drug conjugate consisting of chimeric CD30 monoclonal antibody [2].

Historically, the median overall survival (OS) for PbL ranged from 8 to 15 months, but recent studies show that the median OS has a wider range [6]. One 2017 study of 135 patients from the LYSA group, 80% of whom received chemotherapy, with a 55% complete response rate, revealed a median OS of 32 months [10]. A 2018 clinical trial of 16 patients found an OS of 62 months after treatment with V-EPOCH: 94% complete response and 6% partial response [9]. A 2021 study that analyzed the survival outcomes of 248 patients treated with chemotherapy in the SEER database found a median survival of 47 months. Additionally, the study suggested that HIV status does not affect the OS of PbL patients [11]. A 2024 analysis of 1822 patients in the SEER database and the NCDB found that the median OS was 58.6 months among treated patients. This analysis also concurred with the 2021 study finding that HIV status did not have a significant impact on the OS of treated PbL patients [12].

Information about the impact of fragmented chemotherapy on the treatment outcomes of PbL is scarce in the current literature. To our knowledge, the presented case is one of the few that reports this impact explicitly. The delay in treatment and incomplete chemotherapy cycles of the presented patient culminated as a result of elopement, outpatient appointments, and incarceration. Despite this, radiographic evidence of tumor shrinkage from 8.1 cm to 3.5 cm was observed over 13.4 months, but it was the result of sporadic, rather than continuous, treatment. This case highlights that even partial treatment can abate the disease progression of PBL, but complete remission is unlikely in the absence of continuity.

Most patients present at an advanced stage due to PbL’s progressive nature and the variable presence of B symptoms, similar to the patient presented here [7]. In general, the prognosis for patients does not change regarding sex, lymphoma stage, HIV status, CD4+ count, viral load, EBV status, or primary site involvement [8,11].

## Conclusions

The presented case illustrates a paucity in the existing literature, as most published studies of PbL focus on patients who receive chemotherapy at regular intervals. The initial presentation with a gradually enlarging mass, without systemic symptoms, contributed to a delayed diagnosis. Treatment was further hindered by the patient’s elopement and repeated non-adherence to follow-up. Although a partial response was achieved with four inpatient cycles of R-EPOCH, remission could not be achieved. This case highlights the complexity of managing PbL, particularly in patients with inconsistent engagement with care.
